# Psychometric evaluation of the Malay version of the Montgomery- Asberg Depression Rating Scale (MADRS-BM)

**DOI:** 10.1186/s12888-015-0587-6

**Published:** 2015-08-19

**Authors:** Anne Yee, Abdul Rahim Mat Yassim, Huai Seng Loh, Chong Guan Ng, Kit-Aun Tan

**Affiliations:** 1 Department of Psychological Medicine, University of Malaya Center for Addiction Sciences (UMCAS), Faculty of Medicine, University of Malaya, 50603 Kuala Lumpur, Malaysia; 2 Department of Malaysian Languages and Applied Linguistics, Faculty of Languages and Linguistics Building, University of Malaya, 50603 Kuala Lumpur, Malaysia; 3 Clinical Academic Unit (Family Medicine), Newcastle University Medicine Malaysia, No. 1, Jalan Sarjana 1, Kota Ilmu, EduCity@Iskandar, 79200 Nusajaya, Johor Malaysia; 4 Department of Psychiatry, Faculty of Medicine and Health Sciences, Universiti Putra Malaysia, UPM, 43400 Serdang, Selangor Malaysia

## Abstract

**Background:**

This study examines the psychometric properties of the Malay version of the Montgomery-Ǻsberg Depression Rating Scale (MADRS-BM).

**Methods:**

A total of 150 participants with (*n* = 50) and without depression (*n* = 100) completed the self-rated version of the Montgomery-Ǻsberg Depression Rating Scale (MADRS-S), the Malay versions of the MADRS-BM, the Beck Depression Inventory-II (BDI-II-M), the General Health Questionnaire-12 (GHQ-12), and the Snaith-Hamilton Pleasure Scale (SHAPS-M).

**Results:**

With respect to dimensionality of the MADRS-BM, we obtained one factor solution. With respect to reliability, we found that internal consistency was satisfactory. The scale demonstrated excellent parallel form reliability. The one-week test-retest reliability was good. With respect to validity, positive correlations between the MADRS-BM, BDI-II-M, and the GHQ and negative correlation between the MADRS-BM and SHAPS-M provide initial evidence of MADRS-BM’s concurrent validity. After adjusting for age, gender, ethnicity, educational level, and marital status, individuals with depression significantly reported higher MADRS-BM scores than did individuals without depression. Hence, there is additional evidence for concurrent validity of the MADRS-BM. Cut-off score of 4 distinguished individuals with depression from individuals without depression with a sensitivity of 78 % and a specificity of 86 %.

**Conclusions:**

The MADRS-BM demonstrated promising psychometric properties in terms of dimensionality, reliability, and validity that generally justifies its use in routine clinical practice in Malaysia.

## Background

To study treatment efficacy, researchers often rely on the use of clinician-rated instruments [1]. Clinician-rated instruments like the Montgomery-Åsberg Depression Rating Scale (MADRS) have been widely used to assess depression [2].

The MADRS is a popular scale because of its high inter-rater reliability and high sensitivity to detect changes in treatment effects [2]. Due to these features, the MADRS has been widely used in mood disorders studies [3–5]. However, the MADRS has recently received increased scrutiny due to rising rate of unsuccessful clinical trials [6]. As reported in the clinical trials, poor inter-rater reliability and rater bias are two common shortcomings associated with clinician-rated scales like the MADRS. Due to these shortcomings, clinical assessment pertaining to depression severity is a subject of debate [7]. The robustness of clinical findings is also questionable [7]. To address this research gap, the MADRS-S, a 9-item self-report measure of depression, was developed [8]. Participants rate items on a 4-point Likert scale ranging from 0 (*no depressive symptoms*) to 3 (*worst depressive symptoms*). Possible score ranges from 0 to 27, with higher scores indicating greater symptom severity. The MADRS-S has been found to have a high degree of concordance with the clinician-rated MADRS and demonstrates adequate reliability (alpha = 0.84; intraclass correlation coefficient, ICC = 0.78) [9].

Although in Malaysia, there are a few scientific attempts devoted to validate depression scales such as the Malay versions of the Beck Depression Index (BDI) [10], Beck Depression Index, Second Edition (BDI-II) [11], and the Depression Anxiety and Stress Scales (DASS) [12]. In the case of the Malay version of the BDI, the scale has been validated in a depression sample [10] and has resulted two major revisions—the authors removed four items which have low sensitivity in identifying typical depressive symptoms. Therefore, identification of depressive symptoms in psychiatric samples using the Malay version of the BDI may be prohibited. In the case of the Malay versions of the BDI-II and DASS, the scales have only been validated in specific samples (e.g., men with urological problems, postpartum women, and infertile couples [10, 11]).

The Malay versions of the BDI, BDI-II, and DASS are multidimensional scales. Specific items from these multidimensional scales could not yield a theoretically sound composite score [13], reducing their sensitivity in detecting changes in depression severity [7]. Unlike the aforementioned scales, the MADRS-S is characterized by a single domain and has good sensitivity in detecting changes in depression and in tracing differential effects of drug on placebo/treatment groups [7]. To the best our knowledge, the Malay version of the MADRS-S has not yet been validated. Therefore, the purpose of this study was to examine the psychometric properties of the Malay version of the Montgomery-Åsberg Depression Rating Scale (MADRS-BM).

## Methods

### Study design

#### Stage 1: Early development of the MADRS-BM

We obtained permission from the original author of the MADRS, Stuart M. Montgomery, for conducting this study. A copy of permission letter was sent to the editor of this journal. The scale was translated from English to Malay in parallel by two bilingual clinical psychologists, whereas a bilingual language expert performed the back-translation. Discrepancies between the original version and the back translation were resolved through discussion and adjustments were made, where necessary. In Stage 1, we finalized the initial version of the MADRS-BM with an expert panel of psychiatrists and family physicians.

#### Stage 2: Refinement of the MADRS-BM

We pilot-tested the initial version of the MADRS-BM using 10 native Malay-speaking nurses to identify any flaws in terms of wording. We noted any words that were considered unsuitable or inappropriate. The scale was also reviewed by a psychiatric consultant, who has vast experience in clinical research, to ensure satisfactory face, semantic, criterion, and conceptual equivalences. In Stage 2, we redefined the Malay version of the MADRS-BM.

#### Stage 3: Evaluation of the MADRS-BM

## Participants and procedure

The study was conducted from September until December 2013 at Psychiatric Outpatient Clinic, University Malaya Medical Centre. The study protocol was approved by the Medical Ethics Committee (MEC) of the University Malaya Medical Centre. For the purposes of the study, we recruited individuals with and without depression. Criteria to select individuals with depression include: (a) subjects who were diagnosed with major depressive disorder (the first author who is a trained clinical psychiatrist confirmed the diagnoses using the Diagnostic and Statistical Manual of Mental Disorders, 4th Edition, Text Revision (DSM-IV-TR [14]),(b) subjects who had no other major psychiatric illnesses or psychoses, (c) subjects who are capable of understanding and reading Malay or English, (d) subjects who are 18 or above, and (e) subjects who gave consent with regard to participation of this study. Individuals without depression were medical workers from the University Malaya Medical Centre. Their participation was based on the criteria as indicated above with the exception of (b). Based on subject to ratios of 5:1, it is statistical appropriate to include 45 individuals with depression and 90 individuals without depression, given that the MADRS-BM has nine items [15]. However, to avoid attrition, we decided to recruit 50 individuals with depression and 100 individuals without depression. For data collection, we identified the eligible subjects and explained research procedure to them. After we sought their written consent, we then distributed a self-administered questionnaire. To obtain test-retest reliability of the MADRS-BM, we invited the subjects to complete the scale again after one week.

### Self-administered questionnaire

#### Socio-demographic information

Participants were invited to provide their socio-demographic information such as age, gender, ethnic group, marital status, educational level, religion, and employment status.

#### The Malay version of the Beck Depression Inventory-Second Edition (BDI-II-M)

The BDI-II-M is a 21-item self-report measure of depression based on a 2-week time period [11]. Participants rated items based on a 4-point Likert Scale ranging from 0 (*no depressive symptoms*) to 3 (*worst depressive symptoms*). Higher scores indicate greater depression. As demonstrated in previous study, the scale demonstrated high internal consistency (alpha = 0.89) and split-half reliability (unequal length Spearman Brown = 0.84) [11].

#### The Malay version of the Snaith-Hamilton Pleasure Scale (SHAPS-M)

The SHAPS-M is a 14-item self-report measure of hedonic experience encompassing interest/pastimes, social interaction, sensory experience, and food/drink. Participants rated items based on a 4-point Likert scale ranging from 1 (*definitely disagree*) to 4 (*definitely agree*) [16]. Lower scores indicate greater hedonic experience. The scale exhibited excellent internal consistency (alpha = 0.96), concurrent validity, and parallel form reliability (ICC = 0.65) in previous study [17].

#### The Malay version of the General Health Questionnaire-12 (GHQ-12)

The Malay version of the GHQ-12 is a 12-item self-report measure of current mental health. Participants rated items based on a 4-point Likert scale ranging from 0 (*always*) to 3 (*never*) for positive items and ranging from 3 (*always*) to 0 (*never*) for negative items. Higher scores indicate greater symptom severity. As shown in previous study, the scale has good internal consistency (alpha = 0.85) [18].

#### The self-rated version of the Montgomery-Ǻsberg Depression Rating Scale (MADRS-S)

The English version of the MADRS-S is a 9-item self-report measure of depression. Participants rated items on a 4-point Likert scale ranging from 0 (*no depressive symptoms*) to 3 (*worst depressive symptoms*). Higher scores indicate greater symptom severity. As demonstrated in previous study, the scale has good parallel form reliability (ICC = 0.78) and adequate reliability (alpha = 0.84) [9].

#### The Malay version of the Montgomery-Ǻsberg Depression Rating Scale (MADRS-BM)

The Malay version of the MADRS-BM is a 9-item self-report measure of depression. Both the MADRS-BM and the MADRS-S are identical in terms of scoring and interpretation as mentioned above.

## Statistical analyses

Data analyses were completed with the use of Statistical Package for the Social Sciences version 20.0 (SPSS, Chicago, IL, USA). Baseline characteristics pertaining to participants were computed using descriptive statistics. To establish dimensionality of the MADRS-BM, we performed principal component analysis. We used Cronbach’s alpha to provide an indication of internal consistency. We also assessed the homogeneity of the scales by calculating correlation coefficients between items and total scores, if an item was deleted. To examine the parallel form reliability between the MADRS-BM and MADRS, and the one week test-retest reliability of the MADRS-BM, we calculated the ICCs. In establishing concurrent validity, we examined correlations between the MADRS-BM and other measures (BDI-II-M, GHQ-12, and SHAPS-M) with Spearman’s test. To examine whether individuals with and without depression would differ significantly in terms of the MADRS-BM scores, we performed analysis of covariance (ANCOVA), while controlling for age, gender, ethnicity, , marital status, and educational level. The optimal MADRS-BM cut off score for individuals with depression was determined on the co-ordinate points as indicated in the receiver operating characteristic (ROC) analysis; we then obtained the rates of sensitivity and specificity

## Results

Table 1 shows demographic information across participants with and without depression. We recruited 50 participants with depression (50 % male, 50 % female) and 100 participants without depression (28 % male, 72 % female).

**Table 1:** Socio-demographic characteristics

Variables	Cases		Total, *N* = 150 (%)	*P* value
	With Depression *N* = 50(%)	Without Depression *N =* 100 (%)		
Age (mean ± SD)	47.16 ± 14.4	29.09 ± 10.53	35.11 ± 14.68	<.01
Gender				<.05
Male	25 (50.0)	28 (28.0)	53 (35.3)	
Female	25 (50.0)	72 (72.0)	97 (64.7)	
Marital Status				<.01
Single	13 (26.0)	64 (64.0)	77 (51.3)	
Married	32 (64.0)	35 (35.0)	67 (44.7)	
Divorced	1 (2.0)		1 (0.7)	
Widowed	4 (8.0)	1 (1.0)	5 (3.3)	
Ethnicity				<.01
Malay	15 (30.0)	70 (70.0)	85 (56.7)	
Chinese	20 (40.0)	23 (23.0)	43 (28.7)	
Indian	12 (24.0)	7 (7.0)	19 (12.7)	
Others	3 (6.0)		3 (2.0)	
Religion				<.01
Islam	16 (32.0)	71 (71.0)	87 (58.0)	
Christianity	9 (18.0)	5 (5.0)	14 (9.3)	
Buddhism	15 (30.0)	15 (15.0)	30 (20.0)	
Hinduism	7 (14.0)	7 (7.0)	14 (9.3)	
Others	3 (6.0)	2 (2.0)	5 (3.3)	
Educational Level				<.05
Primary	2 (4.0)		2 (1.3)	
Secondary	19 (38.0)	21 (21.0)	40 (26.7)	
Tertiary	29 (58.0)	79 (79.0)	108 (72)	
Employment Status				0.941^a^
Employed	24 (48.0)	54 (54.0)	78 (52.0)	
Unemployed	26 (52.0)	46 (46.0)	72 (48.0)	

*Note:* ^a^Not significant

### Dimensionality of the MADRS-BM

Bartlett’s test of sphericity was significant (*p* < .01) and the Kaiser-Meyer-Olkin measure of sampling adequacy for the MADRS-BM was 0.93, indicating that the sampling adequacy was meritorious [19]. A single factor was extracted using the principle component approach (eigenvalue >1.00), which accounted for 61.3 % of the total variance. Likewise, as indicated by the scree plot, a single predominant factor was displayed. Taken together, the MADRS-BM contained only a single construct measuring individuals’ psychological state.

#### Reliability

The MADRS-BM exhibited good internal consistency (alpha = 0.78). All the items had corrected item-total correlations that were 0.7 or above. Removal of items, if any, would not increase the alpha value (see Table 2). The parallel form reliability between the MADRS-S and the MADRS-BM was excellent (ICC = 0.98, *p* < .01). The scale demonstrated good one-week test-retest reliability (ICC = .88, *p* < .01).

**Table 2:** Corrected item-total correlations and Cronbach’s alpha values if an item is deleted in the MADRS-BM

MADRS-BM	Scale mean if item is deleted	Scale variance if item is deleted	Corrected item-total correlation	Cronbach’s alpha if item is deleted
Mood	7.02	75.55	0.79	0.75
Feelings of unease	6.69	75.53	0.78	0.75
Sleep	6.68	78.25	0.57	0.76
Appetite	7.06	78.14	0.62	0.76
Ability to concentrate	6.82	75.31	0.77	0.75
Initiative	6.87	75.55	0.80	0.75
Emotional involvement	6.99	75.68	0.82	0.75
Pessimism	6.83	75.13	0.80	0.75
Zest for life	7.08	77.08	0.76	0.76
Total score	3.66	21.32	1.00	0.91

*Note: MADRS-BM* = Malay version of the Montgomery-Ǻsberg Depression Rating Scale

#### Validity

The MADRS-BM was significantly and positively correlated with the BDI-II-M (*p* < .01) and the GHQ (*p* < .01) scores, but the scale was significantly and negatively correlated with the SHAPS-M (*p* < .01). Therefore concurrent validity of the MADRS-BM was established (see Table 3).

**Table 3:** Spearman’s correlations (*r*) among the (MADRS-BM), BDI-II, GHQ-12, and SHAPS-M

	MADRS-BM	BDI-II-M	GHQ-12	SHAPS-M
MADRS-BM	1.000	0.779**	0.584**	−0.398**
BDI-II		1.000	0.659**	−0.393**
GHQ-12			1.000	−0.439**
SHAPS-M				1.000

*Note: MADRS-BM* = Malay version of the Montgomery-Ǻsberg Depression Rating Scale; *BDI-II* = Beck Depression Index, second version; *GHQ-12* = General Health Questionnaire-12; *SHAPS* = Snaith-Hamilton Pleasure Scale.

*P < .05, **P < .01

**Table 4:** Comparison between individuals with and without depression with regard to the MADRS-BM total scores and item scores

The MADRS-BM	The MADRS-BM mean scores		Mean difference	Adjusted mean difference^a^	*P* value
	Mean (SD)			(95 % CI)	
	Case	Control			
Mood	0.83	0.45	0.78	0.68	<.001
	(0.91)	(0.23)		(0.43, 0.92)	
Feelings of unease	1.11	0.39	0.72	0.74	<.001
	(0.79)	(0.45)		(0.48, 1.01)	
Sleep	0.97	0.16	0.80	0.73	<.001
	(0.80)	(0.32)		(0.49, 0.97)	
Appetite	0.66	0.07	0.59	0.39	<.001
	(0.90)	(0.21)		(0.16, 0.63)	
Ability to concentrate	0.96	0.27	0.68	0.70	<.001
	(0.87)	(0.46)		(0.42, 0.98)	
Initiative	0.90	0.23	0.66	0.67	<.001
	(0.84)	(0.41)		(0.42, 0.93)	
Emotional involvement	0.88	0.05	0.82	0.83	<.001
	(0.84)	(0.19)		(0.62, 1.04)	
Pessimism	1.00	0.23	0.76	0.78	<.001
	(0.86)	(0.39)		(0.52, 1.04)	
Zest for life	0.66	0.04	0.62	0.65	<.001
	(0.85)	(0.16)		(0.42, 0.87)	
Total score	7.97	1.51	6.46	6.21	<.001
	(5.70)	(1.39)		(4.71, 7.70)	

*Note:* ^a^adjusted for age, gender, ethnicity, educational level, and marital status

After adjusting for age, gender, ethnicity, educational level, and marital status, individuals with depression (*M* = 7.97, *SD* = 5.70) significantly reported higher MADRS-BM scores than did individuals without depression (*M* = 1.51, *SD* = 1.39) (Table 4). Our findings found additional evidence for concurrent validity of the MADRS-BM.

The area under the receiver operating characteristic curve (i.e., the AUC) was 0.91 (95 % CI = 0.86-0.96). The optimal cut-off score to distinguish individuals with depression from individual without depression was ≥ 4 with a sensitivity of 78 % and a specificity of 86 %.

## Discussion

Our current findings show that the MADRS-BM has good internal consistency with an alpha value of 0.70. This result is comparable to the properties of the clinician-rated MADRS (alpha = 0.70) [2]. Also comparable to the original version of the MADRS, the MADRS-BM demonstrated good parallel form reliability (ICC = 0.98) and one-week test-retest reliability (ICC = 0.88) [9]. The present findings reveal that the MADRS-BM is at least equivalent, if not better, to the MADRS as an assessment tool for depression. In terms of dimensionality, our findings revealed a single factor that accounted a large proportion of the variance in MADRS-BM. In line with previous studies, its factor structure was similar to that of the MADRS [7, 9].

We also examined the concurrent validity of the the MADRS-BM by linking the MADRS-BM with the BDI-II-M, GHQ-12, and SHAPS-M. Positive correlations between the MADRS-BM, BDI-II-M, and GHQ and negative correlation between the MADRS-BM and SHAPS-M provide initial evidence of MADRS-BM’s concurrent validity. Additional evidence for concurrent validity of the MADRS-BM was reported. After adjusting for some socio-demographic information, individuals with depression significantly reported higher MADRS-BM scores as compared to individuals without depression.

In this study, the cut off score for the MADRS-BM was 4, which is lower than the recommended score of 5, as suggested by the original MADRS. One possible explanation is that the current version of the MADRS-BM is a self-rated scale—participants tend to underrate or underestimate their symptoms. Even though the cut off score was lower than that of the MADRS, the MADRS-BM’s sensitivity was greater than that of the MADRS.

A few limitations of this study warrant consideration. Firstly, given the cross-sectional nature of this study, we were unable to rule out the causal factors of depression. Likewise, we were unable to assess the predictive validity of the MADRS-BM. Secondly, our sample was recruited from an outpatient clinic in a tertiary hospital using convenience sampling; thus we raised concern over generalizability as one possible limitation. Lastly, some clinical features such as the severity of depression and the types of antidepressants being used by the patients were not documented in the current study. The presence of such clinical features could affect the MADRS-BM scores as reported by participants.

## Conclusion

In spite of these limitations, the MADRS-BM demonstrated promising psychometric properties in terms of dimensionality, reliability, and validity that generally justifies its use in routine clinical practice in Malaysia. In order to further establish its psychometric properties, future diagnostic studies using the standards for reporting of diagnostic accuracy (STARD) criteria are recommended.

## Endnotes

This is a requirement for online studies made by the local ethics committee.
